# Plasma‐Activated Hydrogels for Microbial Disinfection

**DOI:** 10.1002/advs.202207407

**Published:** 2023-03-16

**Authors:** Jinkun Chen, Zifeng Wang, Jiachen Sun, Renwu Zhou, Li Guo, Hao Zhang, Dingxin Liu, Mingzhe Rong, Kostya (Ken) Ostrikov

**Affiliations:** ^1^ State Key Laboratory of Electrical Insulation and Power Equipment Centre for Plasma Biomedicine Xi'an Jiaotong University Xi'an City 710049 People's Republic of China; ^2^ School of Chemistry and Physics Centre for Materials Science, and Centre for Biomedical Technologies Queensland University of Technology (QUT) Brisbane QLD 4000 Australia

**Keywords:** antibacterial effect, long‐term activity, plasma‐activated hydrogel, reactive species

## Abstract

A continuous risk from microbial infections poses a major environmental and public health challenge. As an emerging strategy for inhibiting bacterial infections, plasma‐activated water (PAW) has proved to be highly effective, environmental‐friendly, and non‐drug resistant to a broad range of microorganisms. However, the relatively short lifetime of reactive oxygen and nitrogen species (RONS) and the high spreadability of liquid PAW inevitably limit its real‐life applications. In this study, plasma‐activated hydrogel (PAH) is developed to act as reactive species carrier that allow good storage and controlled slow‐release of RONS to achieve long‐term antibacterial effects. Three hydrogel materials, including hydroxyethyl cellulose (HEC), carbomer 940 (Carbomer), and acryloyldimethylammonium taurate/VP copolymer (AVC) are selected, and their antibacterial performances under different plasma activation conditions are investigated. It is shown that the composition of the gels plays the key role in determining their biochemical functions after the plasma activation. The antimicrobial performance of AVC is much better than that of PAW and the other two hydrogels, along with the excellent stability to maintain the antimicrobial activity for more than 14 days. The revealed mechanism of the antibacterial ability of the PAH identifies the unique combination of short‐lived species (^1^O_2_, ∙OH, ONOO^−^ and O_2_^−^) stored in hydrogels. Overall, this study demonstrates the efficacy and reveals the mechanisms of the PAH as an effective and long‐term disinfectant capable of delivering and preserving antibacterial chemistries for biomedical applications.

## Graphical abstract

This work demonstrates the possibility of plasma activated hydrogels being an effective disinfectant with great prospects for the delivery and long‐term preservation of reactive plasma chemistries for preventing bacterial infections and related biomedical applications.

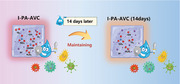

## 1 Introduction

Microbial infections carry a high risk of mortality, and the often excessive use of commercial antibiotics has led to the emergence of multi‐antibiotic‐resistant pathogens that exacerbate microbial infections, which pose a major challenge to the environment and public health.^[^ ^1^, ^2^, ^3^ ^]^ Cold atmospheric pressure plasma (CAP) is as a promising broad‐spectrum biocidal agent due to a favorable combination of physical effects and reactive chemical species such as reactive oxygen species (ROS), reactive nitrogen species (RNS), charged particles, ultraviolet photons, and transient electric fields.^[^ ^4^, ^5^, ^6^, ^7^ ^]^ The CAP finds more and more applications in wound healing, rapid hemostasis, cancer treatment, and other fields with enormous prospects for medical therapies.^[^ ^8^, ^9^ ^]^ More recently, CAP reacting with liquids for the preparation of plasma‐activated water (PAW) has emerged as a new cost‐effective and environmentally benign biological agent for inhibiting bacterial and viral infections.^[^ ^10^, ^11^, ^12^ ^]^ Interactions of the plasma‐generated species, electric fields, and light with water generate a rich variety of liquid‐phase reactive species (such as H_2_O_2_, NO_2_^−^, ∙OH, ^1^O_2_, O_2_^−^, and ONOO^−^) through cascades of gas–liquid and liquid–liquid reactions.^[^ ^13^, ^14^, ^15^ ^]^ However, the relatively short lifetime of reactive oxygen and nitrogen species (RONS) and the limited viscosity of PAW limit the translation of this multipurpose treatment into real‐life applications. Although different solutions including water, phosphate buffer solution, and cell media^[^ ^16^, ^17^ ^]^, have been investigated with a general aim to expand the application area of CAP, the relatively short lifetime of reactive oxygen and nitrogen species (RONS), and the limited viscosity of PAW limit the translation of this multipurpose treatment into real‐life applications.

Here we develop an effective and innovative solution based on hydrogels which are more advanced, practical, and functional model materials than commonly used biological liquids (i.e., water, phosphate buffer solution and cell culture) due to their good water storage properties, water absorption performance, and excellent biocompatibility.^[^ ^18^, ^19^, ^20^, ^21^, ^22^ ^]^ Conventional hydrogels for antibacterial studies are generally loaded with antibiotics, antimicrobial peptides/polymers, or novel metal nanomaterials^[^ ^23^, ^24^, ^25^, ^26^, ^27^, ^28^, ^29^, ^30^, ^31^ ^]^, but they face several challenges (Table S1, Supporting Information). Recently, hydrogels directly or indirectly treated by plasmas, termed plasma‐activated hydrogels (PAHs), have been developed to act as reactive species carriers for biomedicine.^[^ ^32^, ^33^, ^34^ ^]^ PAHs not only have the characteristics of a gel‐like complex physical structure and good rheological properties, but also can preserve plasma chemical species for a long time and release reactive species to the diseased site slowly.^[^ ^35^, ^36^, ^37^, ^38^, ^39^, ^40^ ^]^ PAHs have more excellent antimicrobial properties compared to the existing antimicrobial hydrogels (Figure S1, Supporting Information), due to the complex chemical components and a large variety of polymer chains contained in hydrogels, interactions between the plasma‐generated reactive species and hydrogels can lead to cross‐linked structural changes or functionalization of the hydrogel surface, with many types of functional groups that can be introduced.^[^ ^33^, ^41^, ^42^, ^43^ ^]^ We hypothesize that different types of hydrogels and different plasma processing methods may result in different physicochemical properties and functionalities of PAHs, which may further improve their biological benefits and applications through the as yet unknown physicochemical mechanisms.^[^ ^44^, ^45^, ^46^, ^47^ ^]^ And this cross‐sectional comparison of different types of hydrogels and their physicochemical properties after being activated by plasma which can be a reference for PAH methods is still lacking relatively well studied.

On the other hand, although PAHs have been demonstrated to have antimicrobial and sterilizing reactivity for inhibiting bacterial infections^[^ ^48^ ^]^, the commonly used coating plate or permeation methods are difficult to calculate their sterilization efficiency. In some cases, PAHs were prepared by mixing the intermediate PAW and gel powders^[^ ^34^, ^35^ ^]^, so it is also necessary to compare the biological activity of PAHs and PAW. Moreover, the mechanisms that underpin the stable biological activity of PAH have not been fully understood, especially with respect to the contribution of different reactive species to its biochemical and biological activity. Therefore, comparative investigations of the changes in the properties of different hydrogels after the plasma treatment and revealing the reactive species which “activate” the hydrogels to achieve the desired biological effects, are crucial to progress the development and applications of PAH technologies.

**Figure 1:**
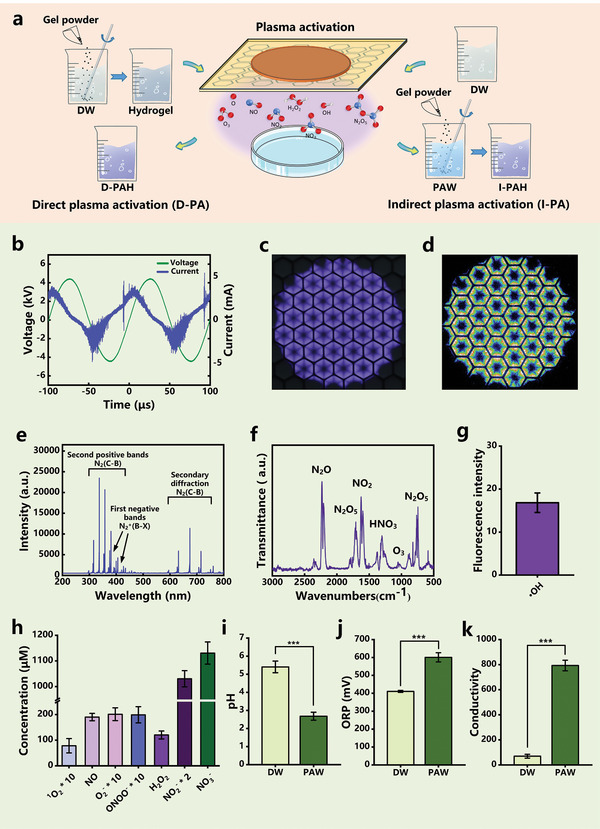
Preparation of plasma‐activated hydrogel (PAH), generation, and detection of gas–liquid phase reactive species responsible for biological activity. a) Direct plasma activation (D‐PA) and indirect plasma activation (I‐PA) of hydrogels. b) Waveforms of discharge voltage and current. c) Discharge image of surface plasma. d) Intensified CCD (ICCD) image of surface plasma. e) Emission spectra of surface plasma discharge. f) Fourier infrared spectrometer (FTIR) spectra of surface plasma discharge. g) Concentrations of short‐lived active species in plasma‐activated water (PAW) measured by the enzyme marker. h) Concentrations of long‐lived and short‐lived active species in PAW measured by electron spin resonance (ESR). i) pH of de‐ionized water (DW) and PAW. j) Oxidation–reduction potential (ORP) of DW and PAW. k) Conductivity of DW and PAW. Statistical analysis was performed using *t*‐test (*n* = 3, ^***^ *p* < 0.001).

In this study, three different commonly used hydrogel materials, including hydroxyethyl cellulose (HEC), Carbomer 940 (Carbomer), and ammonium acryloyldimethyl taurate/VP copolymer (AVC), are selected as model bio‐gels to investigate and compare their bactericidal effects after the plasma treatment. To investigate the different characteristics of hydrogels, direct plasma activation (D‐PA) and indirect plasma activation (I‐PA) methods were employed for the PAH preparation (**Figure** **1**a). Fourier infrared spectrometer (FTIR) was used to analyze the structural changes of the hydrogels before and after the plasma treatment. Transformation pathways of reactive species from the gaseous plasma, liquid phase media (PAW), and to the final product (PAH) are elucidated. New insights into the physicochemical properties of PAW and PAHs, including pH, oxidation–reduction potential (ORP), and the concentrations of reactive species inside the hydrogels, are obtained. Sterilization effects of thus‐produced PAHs and PAW were comparatively evaluated, and the role of different reactive species in bacterial inactivation was determined. Finally, the stability of PAH under room temperature was also studied and compared with PAW leading to new insights into the temporal dynamics and underlying mechanisms of the PAH chemistry.

## 2 Results

### 2.1 Characteristics of Gaseous Plasma and PAW

Previous studies have shown that the mode of surface dielectric barrier discharge plasma can be modulated by adjusting the process parameters such as the discharge power, air flow rate, and temperature.^[^ ^49^ ^]^ In this work, using a surface dielectric barrier discharge (S‐DBD) (Figure S2, Supporting Information), the peak– discharge voltage of 9.0 kV and the discharge frequency of 10.0 kHz were set for the plasma ignition (Figure 1b). The discharge power density was controlled at 1.5 W cm^−2^ (Figure S2, Supporting Information), and the plasma discharge was ignited in a confined chamber (Figure 1c). The discharge electric field was concentrated on both sides of the mesh electrode, resulting in the formation of a series of narrow discharge pulses, also known as filamentary discharges (Figure 1d).^[^ ^49^, ^50^ ^]^ Optical emission spectra (OES) were employed to determine reactive gaseous species in gaseous plasmas. Figure 1e shows the optical emission spectrum in the wavelength range from 200 to 800 nm, which is mainly composed of the first negative bands of N_2_^+^(B^2^ $\Sigma_{u}^{+}$ →X^2^ $\Sigma_{g}^{+}$), the second positive bands of N_2_(C^3^Π_u_→B^3^Π_g_), and secondary diffraction of N_2_(C^3^Π_u_→B^3^Π_g_). The generation of N_2_(C^3^Π_u_→B^3^Π_g_) occurs through the electron impact excitation from the ground state N_2_(X^2^ $\Sigma_{g}^{+}$). The first metastable state N_2_(A^2^ $\Sigma_{g}^{+}$) and N_2_^+^(B^2^ $\Sigma_{g}^{+}$) states are produced via direct electron collisions with high‐energy electrons. In this case, the N and O elements in the air are excited to form RONS with biological activity.^[^ ^51^, ^52^ ^]^

The air discharge employed in this work exhibits a hybrid nitrogen “oxide and ozone” mode, which produces a large number of highly reactive nitrogen oxides and oxidative species.^[^ ^6^ ^]^ The main gaseous products generated in this discharge mode are N_2_O, NO, NO_2_, and N_2_O_5_, as well as a small amount of O_3_, since most of the ROS generated in the confined space are reduced by the nitrogen species over a very short period of time (Figure 1f). The presence of water vapor in the ambient air also led to the detection of HNO_3_ in the FTIR spectrum, and the absorption peak of NO_3_^−^ was observed near 1372 cm^−1^ due to the corrosion of the chamber window sheet by HNO_3_. The dissolution of gas‐phase plasma species and their subsequent liquid‐phase reactions produce a unique mixture of highly biochemically reactive chemistries in PAW. The pH of PAW decreases from 5.41 to 2.68 after the 5 min plasma treatment, while the OPR and conductivity increase significantly (Figure 1i–k). The concentrations of aqueous species in PAW, including NO, O_2_^−^, ONOO^−^, H_2_O_2_, NO_2_^−^, NO_3_^−^, ^1^O_2_, and ∙OH (Figure 1g,h). Among them, the concentrations of NO_2_^−^, NO_3_^−^, and NO are much higher than that of O_2_^−^ and H_2_O_2_, due to the dissolution of plasma‐generated nitrogen oxide species.^[^ ^5^, ^6^ ^]^ All these results presented in Figure 1 indicate that the reactive species produced by the plasma are responsible for the reactive PAW chemistries utilized in the subsequent biomedical applications.

### 2.2 Physicochemical Properties of PAH

**Figure 2:**
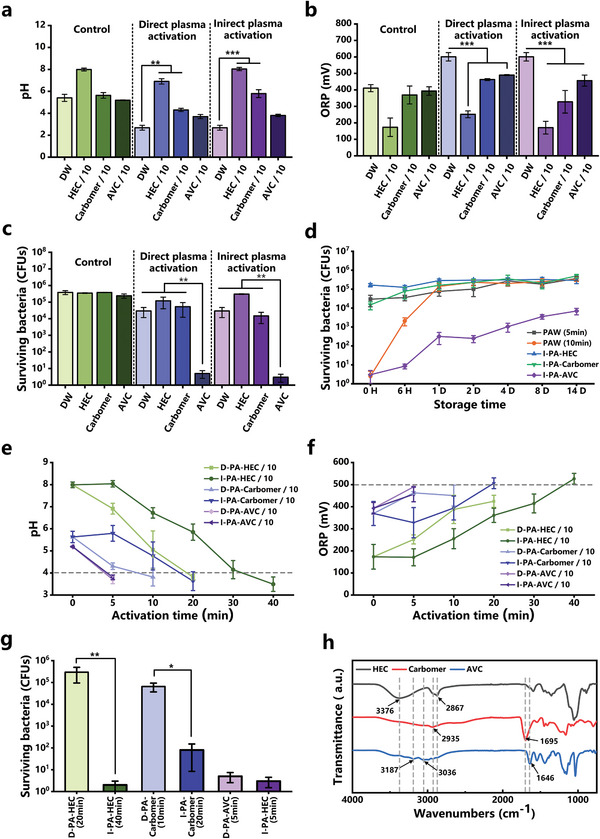
Physicochemical properties and antimicrobial activity of plasma‐activated hydrogel (PAH). a–c) pH, oxidation–reduction potential (ORP) and antimicrobial activity of PAH after direct plasma activation (D‐PA) and indirect plasma activation (I‐PA). d) Antibacterial activity and trends of plasma‐activated water (PAW) (5 min), PAW (10 min), and three PAHs during storage. e,f) Variation of pH and ORP with activation time for hydroxyethyl cellulose (HEC), Carbomer, and acryloyldimethylammonium taurate/VP copolymer (AVC) under two plasma activation methods. g) Antimicrobial activity of HEC, Carbomer, and AVC at similar pH and ORP with two plasma activation methods. h) Fourier infrared spectrometer (FTIR) spectra of HEC, Carbomer, and AVC without the plasma treatment. Statistical analysis was performed using *t*‐test (*n* = 3, ^*^ *p* < 0.05, ^**^ *p* < 0.01, ^***^ *p* < 0.001).

As for the preparation of direct plasma‐activated hydrogel (D‐PAH) and the indirect plasma‐activated hydrogel (I‐PAH), gas‐phase plasma reactive species can penetrate the hydrogel during the direct plasma treatment, or liquid‐phase reactive species can enter PAH by mixing with PAW and gel powder, in which these reactive species may also react with the hydrogel components. To investigate the effects of reactive plasma species on the polymer network of the hydrogels, three hydrogel materials with direct or indirect plasma treatments were characterized by FTIR. As shown in **Figure** **2**h, the absorption spectra of HEC, Carbomer, and AVC showed large differences due to the different chemical structures and components of the polymers, which also indicate their different physicochemical characteristics. The main absorption peaks of all three hydrogels are located in the characteristic region (4000–1300 cm^−1^). Among them, the absorption peaks of HEC were more obvious around 3376 cm^−1^ (O–H stretching vibration) and 2867 cm^−1^ (−CH_2_− stretching vibration); the absorption peaks of Carbomer were more obvious around 2935 cm^−1^ (O−H stretching vibration) and 1695 cm^−1^ (C=O stretching vibration); the absorption peaks of AVC were more obvious around 3187 cm^−1^ (N−H stretching vibration), 3036 cm^−1^ (−C=−H stretching vibration), and 1646 cm^−1^ (−C=C‐stretching vibration). The different functional groups of these gels are also possibly linked with their physicochemical properties and biological activities. However, no significant absorption peak shifts were observed in the FTIR spectra before and after the plasma treatment, suggesting that neither direct nor indirect plasma activation significantly changed the gel surface groups (Figure S4, Supporting Information). In this context, the biological properties of PAH mainly originate from a combination of reactive plasma species and their own surface functional groups.

Previous studies have demonstrated that PAW can be an effective disinfectant for inhibiting a wide range of microorganisms, and its antibacterial activity is directly proportional to ORP value and inversely proportional to pH value.^[^ ^6^ ^]^ Generally, a high ORP value represents a high oxidation capacity and theoretical antibacterial ability of PAW. In this work, the pH and ORP values of de‐ionized water (DW) and three gel samples were tested before and after the plasma treatment. To make the measurements more accurate, these plasma‐activated gels were diluted by 10‐folds to improve their flowability, and their pH and ORP values were determined at room temperature. There is a significant decrease in the pH of DW after the plasma treatment, while the pH decreases in HEC, Carbomer, and AVC are relatively slow due to the different penetration depths of the plasma species and different buffering effects of water and gels (Figure 2a). On the other hand, the pH value of I‐PAHs was higher than that of D‐PAHs. This may be attributed to the fact that the charged particles, UV radiation, and the increase in gas temperature within the discharge chamber (Figure S5, Supporting Information), which can modify the composition, structure, or surface functionality of the gels, resulting in a weakened buffering effect.^[^ ^53^, ^54^ ^]^ In Figure 2b, the ORP increase of DW after the plasma treatment is also higher than that of these three gels, and the ORP of D‐PAHs is much higher than that of I‐PAHs, which is also consistent with the pH change, i.e., the lower the pH value, the higher the ORP in the PAH system. Therefore, PAH has achieved a buffering response of pH and ORP under plasma activation, and we expect to discussing other properties of PAH in the next section.

### 2.3 Sterilization Comparison between PAW and PAH

To evaluate the antimicrobial activity of PAHs, *Staphylococcus aureus* (*S. aureus*) was selected as a model microorganism in this study and the number of surviving colonies on TSB agar plates was counted. Figure 2c shows the sterilization effects of PAW and PAH under different plasma treatment conditions. After 5 min of the plasma treatment, PAW was able to inactivate about an order of magnitude of bacterial cells. Three gels with the plasma treatment showed different antibacterial performances, with the highest sterilization effect of PA‐AVC, reaching a level of about five orders of magnitude. This may be attributed to the fact that the PAH activity is closely related to the composition and structure of the hydrogels. Moreover, the bactericidal effects of D‐PAH and I‐PAH are not very different and are in the same order of magnitude.

Although I‐PAH was prepared using the 5 min‐PAW, the antibacterial activity of I‐PAH was significantly different from that of PAW, while some differences were observed between different gel‐based PAHs. In order to better compare the antibacterial activity of PAW and PAH, the indirect treatment method was employed to prepare different hydrogels. As shown in Figure 2d, a similar antibacterial performance of indirect plasma activated AVC (I‐PA‐AVC) and PAW was achieved when the preparation time of PAW was extended to 10 min. After the storage of 6 h, the bactericidal effect of 10 min‐PAW declined from five orders to two orders of magnitude and its antibacterial activity was lost after 1 day of storage. In contrast, the antibacterial activity of I‐PA‐AVC declined slowly, which can still achieve two orders of magnitude of bacterial inactivation even after 14 days. It is presumed that this is because AVC hydrogels have hydrophilic groups such as ammonium groups, which dissolve into gel‐like dispersions in aqueous solutions (PAW) with a unique spatial structure, when the droplets are covered by fully adsorbed polymer molecules and the reactive species are separated from each other and from external reducing substances, thus reducing their consumption during storage and prolonging the antimicrobial activity.^[^ ^36^, ^37^, ^38^, ^55^ ^]^

In order to further discuss the correlation between the pH and ORP of PAHs and their antibacterial activities, the activation time for the PAH preparation was extended to maintain their pH and ORP values at a similar level (Figure 2e,f). The pH of direct plasma activated HEC (D‐PA‐HEC) (20 min), indirect plasma activated HEC (I‐PA‐HEC) (40 min), direct plasma activated Carbomer (D‐PA‐Carbomer) (10 min), and indirect plasma activated Carbomer (I‐PA‐Carbomer) (20 min) were in the range of 3.0–4.0, and their ORP values were in the range of 400–550 mV, while the activation time for AVC was relatively short for achieving the desired pH and ORP values. Moreover, the activation time of I‐PAHs was much longer than that of D‐PAHs to acquire similar parameters, because RONS are delivered faster by direct plasma and diffuse slowly in the PAW. Figure 2g shows the comparison of the antibacterial performances of PAHs under similar pH and ORP conditions. Clearly, the antibacterial activity of D‐PA‐HEC (20 min) and D‐PA‐Carbomer (10 min) was relatively low, with only 0–1 orders of magnitude of bacterial inactivation, while I‐PA‐Carbomer (20 min) was able to kill more than three orders of magnitude of bacterial cells. Further extension of the plasma activation time can enhance the antibacterial activity of PAHs, even in the case of HEC and Carbomer systems, which may be attributed to the accumulation of reactive species. For example, extending the indirect plasma treatment of HEC for 40 min can achieve around five orders of magnitude of bacterial inactivation. Collectively, the results of antimicrobial effect and pH, ORP curves of PAH presented in Figure 2 indicate that the antibacterial activity of PAHs was directly related to the plasma‐generated species stored in hydrogels rather than their pH and ORP values.

### 2.4 Structure and Elemental Analysis of PAH

**Figure 3:**
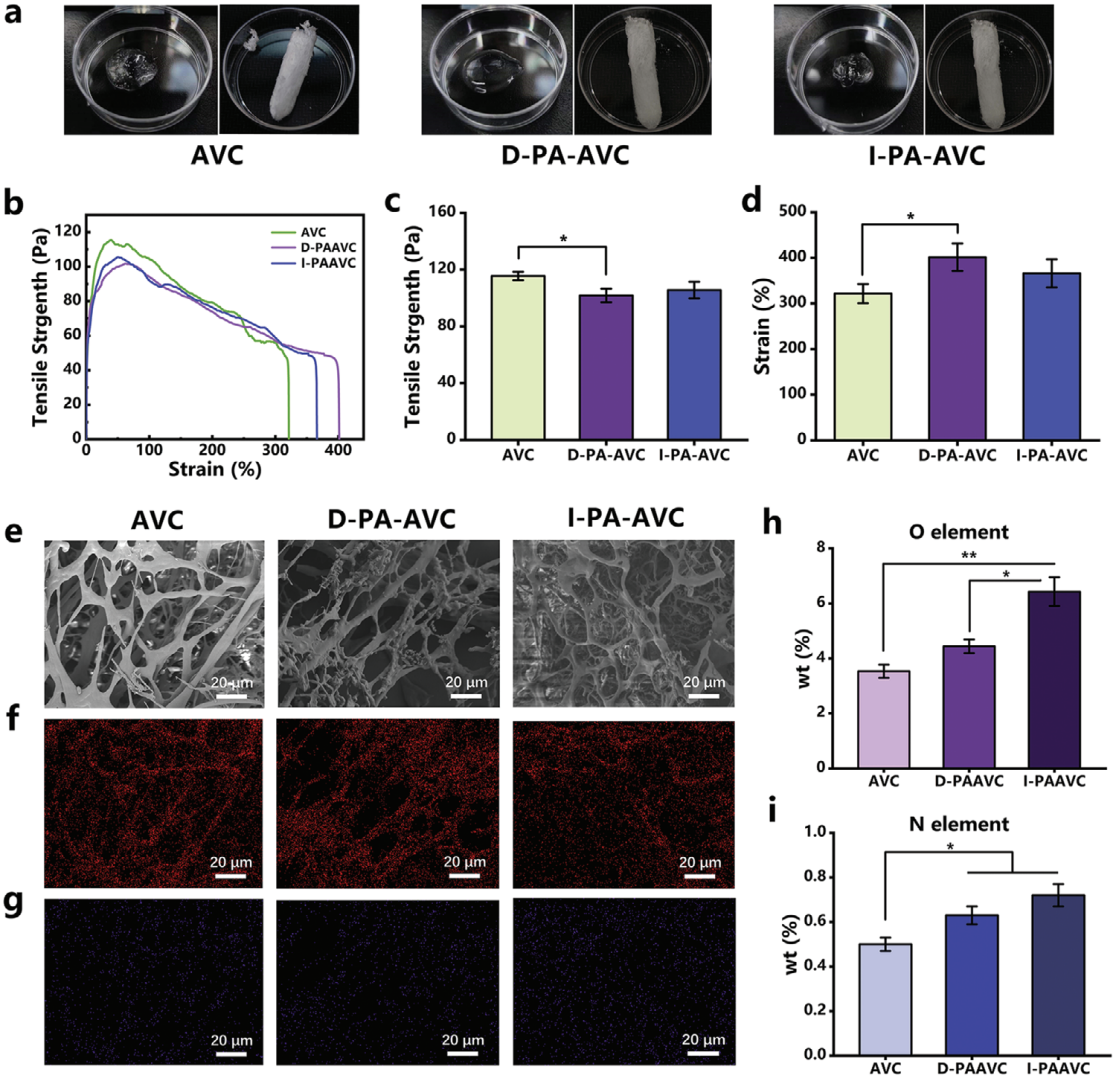
Effect of the plasma activation on the tensile strength, surface morphology, and elemental content of AVC. a) Images and freeze‐dried samples of AVC, D‐PA‐AVC, and I‐PA‐AVC. b) Tensile stress–strain curves. c,d) Fracture strength and tensile fracture strain. e) SEM micrographs of AVC, D‐PA‐AVC, and I‐PA‐AVC. f–g) Distribution of oxygen elements and nitrogen elements in hydrogels. h–i) Analysis of oxygen element content and nitrogen elements. Statistical analysis was performed using *t*‐test (*n* = 3, ^*^ *p* < 0.05, ^**^ *p* < 0.01).

Among these three model bio‐gels, AVC can be effectively activated by the plasma, with better sterilization performance and longer storage time of the reactive species. In this context, this subsection explores the mechanical properties and gel structure of AVC hydrogels with and without the plasma activation. **Figure** **3**a shows images and freeze‐dried samples of AVC, direct plasma activated AVC (D‐PA‐AVC), and I‐PA‐AVC. As shown in Figures 3b–e, the trends of tensile stress–strain curves of the three hydrogels are basically the same. The fracture strength of the plasma‐treated hydrogels slightly decreased, but the tensile fracture deformation increased. This phenomenon may be attributed to the fact that reactive species in the PAH can break the cross‐linked hydrogen bonds in hydrogels. Hydrogen bonding is an important means of physical cross‐linking of hydrogels. When hydrogen bonds are broken, the entanglement between polymer molecular chains will be weakened, and the solution will be diffused easily into the gel network, leading to an increase in the swelling rate.^[^ ^56^, ^57^ ^]^ While the tensile fracture deformation (toughness) of hydrogel is inversely proportional to the cross‐link density^[^ ^58^ ^]^, the intermolecular forces in some hydrogels will be reduced under the action of reactive species. When the cross‐linked polymer is stretched, the chain with the weaker degree of cross‐linking is the first to break. At the same time, the elastic potential energy will be dissipated by other cross‐linked long chains, thus reducing the brittleness of PAH, preventing the occurrence of fracture, and improving the tensile fracture strength.^[^ ^59^, ^60^ ^]^ It is worth mentioning that D‐PA‐AVC has a stronger liquefaction tendency and tensile fracture deformation than I‐PA‐AVC (Figure S6, Supporting Information), since D‐PA‐AVC is subjected to the direct effects of UV, electrons, and reactive species, with the more pronounced disruption of polymer chains.

In order to investigate the tensile strength variation and bioactivity of AVC, we carried out the SEM microscopy and elemental mapping analyses for the AVC and PA‐AVC samples. As observed in the SEM micrographs, the pore sizes of these three gels are similar, while many rounded protrusions appear on the branches of D‐PA‐AVC. Similar pore sizes ensure that the mechanical properties of AVC, D‐PA‐AVC, and I‐PA‐AVC are quite similar.^[^ ^34^ ^]^ However, the direct plasma activation leads to the disruption of cross‐links in the long chains of D‐PA‐AVC hydrogels. As shown in Figure 3e, we observed spherical protrusions on the long chains of D‐PA‐AVC, which may result from long‐chain breakage and recombination, and these structures will lead to a decrease in the tensile strength of D‐PA‐AVC. Meanwhile, the inhomogeneity of the hydrogel long chains makes the hydrogel less brittle, which may be the reason for the enhanced tensile fracture strain of D‐PA‐AVC. Overall, these changes occur as a result of changes in the chemical composition and structure of the long chains of the gel polymer, which are irreversible. As shown in the EDS energy spectrum of the three hydrogels (Figure S7, Supporting Information) and Figure 3f–i, both D‐PA‐AVC and I‐PA‐AVC have higher O and N elemental content than AVC, which is most likely due to the attachment of RONS to the hydrogel structure during the plasma treatment.

## 3 Discussion

**Figure 4:**
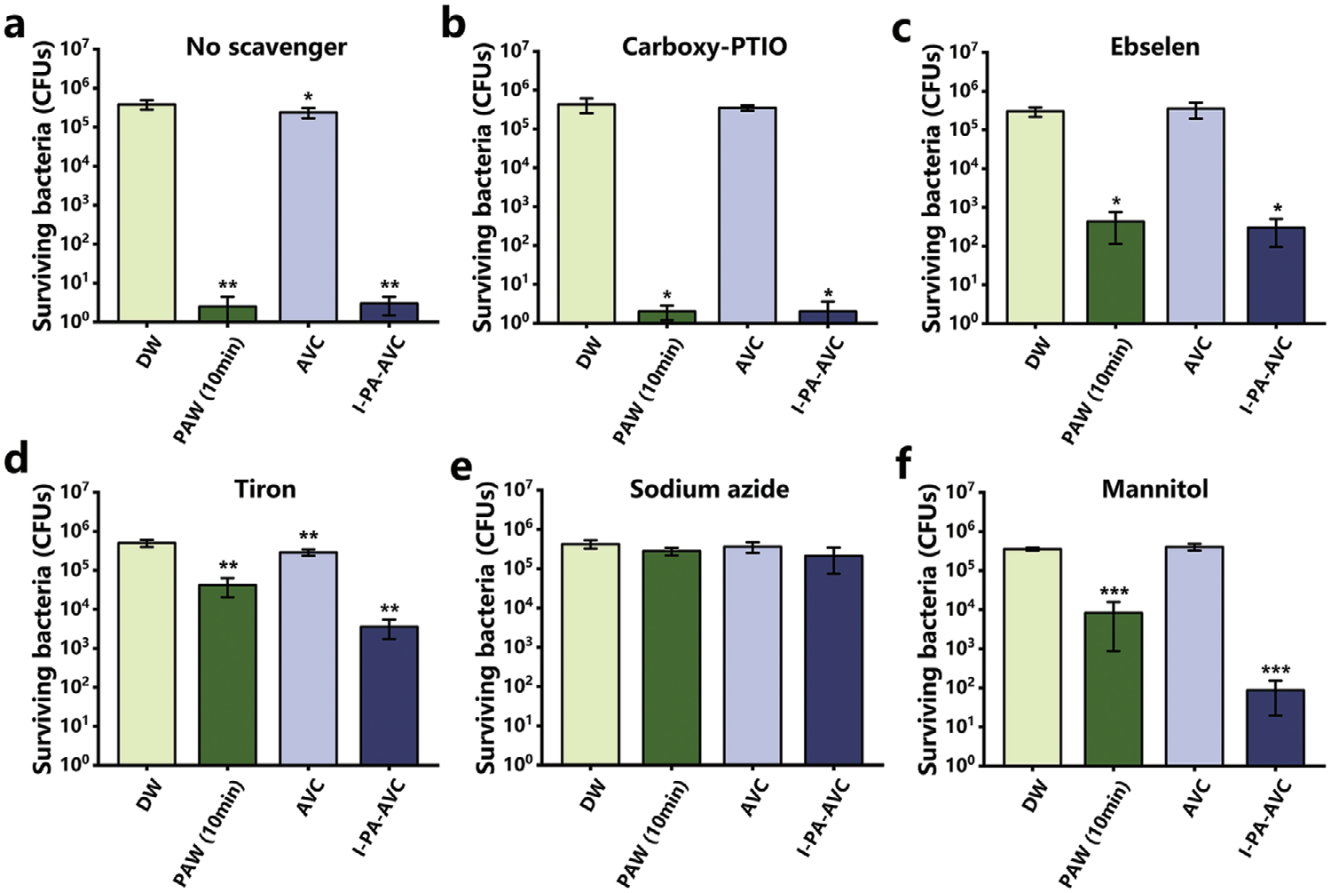
Roles of reactive species in plasma‐activated water (PAW) and I‐PA‐AVC sterilization effects. a) The sterilization effect of PAW (10 min) and I‐PA‐AVC without scavengers. b–f) Effect of scavengers (carboxy‐PTIO, ebselen, tiron, sodium azide, and mannitol) on the sterilization effect of PAW (10 min) and I‐PA‐AVC. Statistical analysis was performed using *t*‐test and the data were compared with the de‐ionized water (DW) (*n* = 3, ^*^ *p* < 0.05, ^**^ *p* < 0.01, ^***^ *p* < 0.001).

It is well known that the antibacterial activity of PAW is inherently related to the rich assortment of highly reactive oxygen and nitrogen species.^[^ ^61^ ^]^ Therefore, can the biological activity of PAW‐made hydrogels also be considered to be“activated”by some key reactive species? In order to elucidate the association of reactive species in I‐PA‐AVC and PAW, we chose to add scavengers to the I‐PA‐AVC hydrogel prepared by PAW (5 min) and PAW (10 min) activated water, respectively. Several different radical scavengers were employed in this study, including mannitol (for ∙OH), sodium azide (for ^1^O_2_ and OH), tiron (for ∙O_2_^−^), ebselen (for ONOO^−^), and carboxy‐PTIO (for ∙NO). As shown in **Figure** **4**, the addition of scavengers to DW and AVC alone did not affect bacterial growth, indicating that the scavengers themselves were not toxic. The scavenging effects of ebselen, tiron, sodium azide, and mannitol on the biological activity of I‐PA‐AVC were obvious, whereas sodium azide almost eliminated all the sterilization ability of I‐PA‐AVC. In contrast, the scavenging effect of carboxy‐PTIO was not significant, which was consistent with the scavenger results obtained in the PAW studies. Collectively, these results indicate that several key ^1^O_2_, ∙OH, ONOO^−^, and O_2_^−^ reactive species are synergistically involved in the activation of gels and responsible for the pronounced sterilization effect of I‐PA‐AVC.

Compared with PAW, PAHs have stronger sterilization activity because of their unique chemical composition and gel structure, which can be better activated by the reactive species.

**Figure 5:**
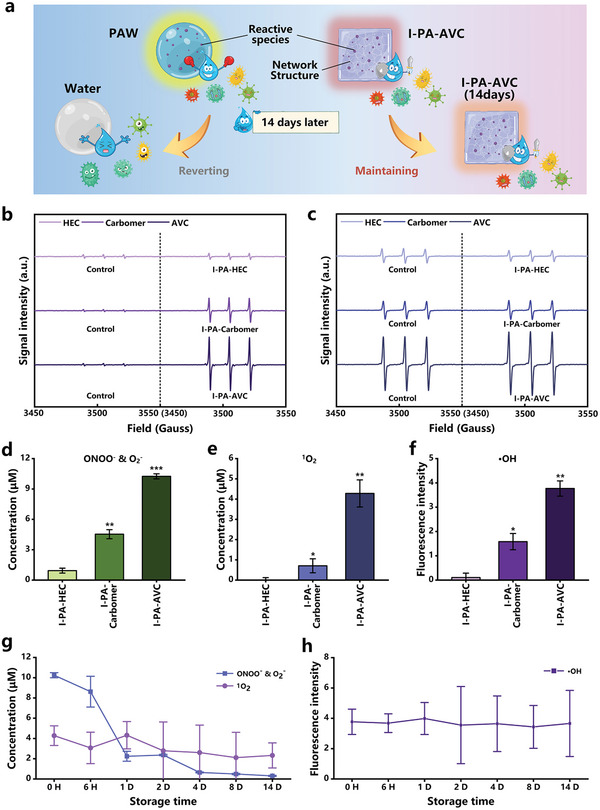
Long‐lasting stable performance of plasma‐activated hydrogels (PAHs). a) Plausible mechanisms of antimicrobial activity of plasma‐activated water (PAW) and PIA‐AVC. b) Electron spin resonance (ESR) spectra of TEMPONE‐H for ONOO^−^ and O_2_^−^ capture in hydroxyethyl cellulose (HEC), Carbomer, and AVC. c) ESR spectra of TEMP for ^1^O_2_ capture in HEC, Carbomer, and AVC. d–f) Concentration of ONOO^−^, O_2_^−^, ^1^O_2_, and ∙OH in I‐PA‐HEC, I‐PA‐Carbomer, and I‐PA‐AVC. g–h) Concentration of ONOO^−^, O_2_^−^, ^1^O_2_, and ∙OH in I‐PA‐AVC during storage. Statistical analysis was performed using *t*‐test and the data were compared with the I‐PA‐HEC (*n* = 3, ^*^ *p* < 0.05, ^**^ *p* < 0.01, ^***^ *p* < 0.001).

Several studies have reported that certain substrate materials in hydrogels (e.g., chitosan and polyacrylamide) have their own biological activities, and even bacterial inhibition.^[^ ^38^, ^62^ ^]^ This may be attributed to the fact that these hydrogels have amine, quaternary ammonium, guanidine, imidazole, and pyridine ionic groups^[^ ^56^ ^]^, and the electrostatic interaction of the ionic groups with the bacterial surface can change the permeability of the bacterial membrane and affect the normal metabolic activity of the bacteria.^[^ ^31^, ^63^ ^]^ However, the antimicrobial ability of hydrogels is relatively weak only by their own groups, and researchers usually use hydrogels loaded with antimicrobial materials and take activation means (such as infrared irradiation) to enhance hydrogels by an antimicrobial order of magnitude or more.^[^ ^64^, ^65^ ^]^ In this study, the AVC hydrogel taken in this study has groups such as ammonium group, amide groups, and pyrrolidone. After the plasma activation, these groups are able to act on the bacteria more efficiently alongside with the reactive species. In contrast, PAW is produced by the plasma treatment of water alone, so the sterilization activity of PAW is relatively lower than that of PA‐AVC. Meanwhile, PAW will lose its sterilization effect after a short period of time, while the network of PAH can restore the reactive species in solution, reducing the loss of their mutual reaction activity and prolonging the antimicrobial activity^[^ ^35^, ^37^ ^]^ and making it a more effective and practical microbial disinfection treatment (**Figure** **5**a). Using the electron spin resonance (ESR) spectrometer and the enzyme marker, we measured the contents of ONOO^−^ O_2_^−^, ^1^O_2_, and ∙OH in I‐PA‐AVC, I‐PA‐Carbomer, and I‐PA‐HEC. As shown in Figure 5b, using the 1‐hydroxy‐2,2,6,6‐tetramethyl‐4‐oxo‐piperidine (TEMPONE‐H) spin trap, we detected the spectra of ONOO^−^ and O_2_^−^, and the signal intensities of all three gels were much higher than those of the control. In Figure 5c, we used 2,2,6,6‐tetramethyl‐4‐piperidone hydrochloride (TEMP) spin trap to capture signals from ^1^O_2_, and the signal intensities of both I‐PA‐AVC and AVC were higher than those of the other two hydrogels. To measure the concentration of reactive species in the hydrogels, we fitted the ESR spectra, integrated them (Figure S8, Supporting Information), and standardized the data presented in Figure 5b,c. As shown in Figure 5d–f, the ONOO^−^, O_2_^−^, ^1^O_2_, and ∙OH concentrations in I‐PA‐AVC are much higher than that in I‐PA‐Carbomer and I‐PA‐HEC, which is consistent with their sterilization results in Figure 2c, confirming the important role of ONOO^−^, O_2_^−^, ^1^O_2_, and ∙OH species in PAH in the sterilization process. It is worth mentioning that although the PAHs were prepared from the mixing of PAW and dry gel powder, the concentration of short‐lived reactive species in the PAHs was lower than that of PAW. This may be due to the consumption by the interaction of reactive species and cross‐linked hydrogen bonds of gel polymers. Thus, the concentration of short‐lived active species in PAHs depends on their reaction with polymer chains and the effect of gel space structure on the adsorption and storage of water molecules. We can speculate that the effect of different types of hydrogels “activated” by the plasma depends on their composition and the chemical structure. Overall, this study provides a new PAH synthesis approach and the best‐performed PA‐AVC sample has a potential to evolve as a new effective microbial disinfectant in real‐world applications. Further, we measured the concentrations of ONOO^−^, O_2_^−^, ^1^O_2_, and ∙OH in I‐PA‐AVC during 14 days of storage (Figure 5g,h). The ^1^O_2_ and ∙OH concentrations remained essentially constant over 14 days, while ONOO^−^ and O_2_^−^ concentrations decreased rapidly after 1 day and were undetectable after 4 days. This trend was similar to the sterilization curve of I‐PA‐AVC in Figure 2d. Regarding the long‐time sterilization effect of I‐PA‐AVC, when it was first prepared, the antimicrobial activity of I‐PA‐AVC was strongest at the initial stage due to the presence of a large number of short‐lived active particles. On the second day, ONOO^−^ and O_2_^−^ disappeared, while ∙OH and ^1^O_2_ species were still present and consistently detected at this time. However, the lack of synergistic effects from ONOO^−^ and O_2_^−^ species somehow reduced the sterilization effect of PAH. The long‐term sterilizing activity of I‐PA‐AVC resulted from the combined action of the residual short‐lived and long‐lived reactive species (Figure S9, Supporting Information). In summary, this section reveals the key role of reactive species in PAH and the reasons for the long‐lasting antimicrobial activity of PAH. However, how these reactive species react with AVC still needs further exploration in future studies.

## 4 Conclusion

To address the limitations of the short lifetime of the reactive plasma species and the viscosity of PAW and PAHs were developed to act as reactive species carriers that allow good storage and controlled slow‐release of RONS to achieve long‐term antibacterial effects. Further, a systematic study was carried out to investigate the interactions of plasma with different types of hydrogels and the synergistic antimicrobial activity of hydrogels. Our results show that the properties of gel materials are the key to the biological activity of thus‐produced PAHs, with the best antibacterial performance achieved in I‐PA‐AVC, along with the long‐term stability to preserve its antimicrobial reactivity for more than 14 days. Further FTIR, pH, and ORP characterizations indicate that AVC had a good buffering performance, and no significant difference was observed in the antibacterial activity of AVC after direct and indirect treatments by the plasma. Reactive species trapping experiments corroborated the mechanisms of the antibacterial ability of PAH involving a combination of short‐lived species (^1^O_2_, ∙OH, ONOO^−^, and O_2_^−^) stored in hydrogels. The proposed PAH technology might offer an effective and environment‐friendly approach with outstanding promise in delivering and preserving reactive plasma chemistries for preventing bacterial infections and related biomedical applications.

## 5 Experimental Section

### Materials

Three model bio‐gels were HEC (ZRNCUN), Carbomer 940 (Carbomer, ZRNCUN), and AVC (ZRNCUN). The scavengers used in this study were mannitol (Sigma–Aldrich), sodium azide (Sigma–Aldrich), tiron (Acros Organics), ebselen (Biochempartner), and carboxy‐PTIO (Sigma–Aldrich). Reactive species detection reagents were disodium terephthalate (TPT, Aladdin), *N*‐(dithiocarbamoyl)‐*N*‐methyl‐D‐glucamine (MGD, Dojindo), TEMP (Aldrich), and TEMPONE‐H (Enzo). The hydrogen peroxide assay kit and the nitrate/nitrite colorimetric kit were from Beyotime Biotech.

### PAH Preparation

Two strategies including direct and indirect plasma activation of the gel materials were employed to prepare PAHs. For the D‐PAH preparation, gel powders were firstly mixed with deionized water and then treated with plasma for 5 min; for I‐PAH, deionized water was initially activated by plasma for 5 min (PAW‐5 min), and then mixed with different hydrogel powders. In the D‐PAH and I‐PAH preparation, 5 mL of DW or PAW was mixed with 0.6% mass fraction of gel powders, shaken, and stood for 10 min and then left to become gel state. To control the variables, the hydrogel treated directly by plasma also stood for 10 min after the plasma discharge.

The PAW and PAH were both prepared using a S‐DBD, which enables a two‐dimensional restriction to the spreading of possible plasma instabilities.^[^ ^66^ ^]^ The S‐DBD structure mainly consisted of a high‐voltage (HV) circular electrode plate (diameter = 42 mm), an Al_2_O_3_ ceramic sheet, and a stainless steel mesh electrode. The power supply (Suman Inc., CTP‐2000K, 500 W) provides a stable sinusoidal AC voltage with a steady frequency of 10 kHz for driving the S‐DBD. A high‐voltage probe (Tektronix, P6015A) and a current probe (Tektronix, P6021) were used to measure the voltage and current, respectively (Figure S1, Supporting Information). An oscilloscope (Tektronix, MDO3054) was employed to record the voltage and current waveforms, and the Lissajous figure of the S‐DBD was plotted to calculate the discharge power (Figure S3, Supporting Information). The peak‐to‐peak discharge voltage of 9.0 kV and the discharge frequency of 10.0 kHz were set for the plasma ignition, with the discharge power density of 1.5 W cm^−2^. Figure 1c,d shows the images of S‐DBD using a digital camera (Nikon D7000) and intensified CCD (ICCD, Teledyne).

### Measurement of RONS in Gas and Liquid Phases

FTIR (Bruker, Tensor II) was used to measure the absorption spectra of gas‐phase reactive species in the range of 500–3000 cm^−1^. The volume of the reaction cell was 56 × 46 × 20 mm. The reaction cell is sealed before the measurement, and the absorption spectrum of air is measured as background. After the plasma discharge, the total absorption spectrum by deducting the background part was the infrared absorption peak of the gas‐phase reactive species.^[^ ^67^ ^]^ Concentrations of reactive species in PAW were measured using a microplate reader (Thermo Science Varioskan Flash Reader) and ESR spectrometer (Bruker). A hydrogen peroxide assay kit was used to measure hydrogen peroxide concentration, and a nitrate/nitrite colorimetric kit was used to measure the nitrate/nitrite concentration. A total of 3 × 10^−3^ m disodium TPT was used to measure the concentration of ∙OH species. NO, ^1^O_2_, O_2_^−^, and ONOO^−^ concentrations were measured by the ESR spectroscopy with the corresponding spin traps, with 100 × 10^−3^ m *N*‐(dithiocarbamoyl)‐*N*‐methyl‐D‐glucamine for trapping NO, 10 × 10^−3^ m TEMP for trapping ^1^O_2_ and 10 × 10^−3^ m TEMPONE‐H for trapping O_2_^−^, and ONOO^−^.

### Physicochemical Properties of PAH and PAW

HEC, D‐PA‐HEC, I‐PA‐HEC; Carbomer, D‐PA‐Carbomer, I‐PA‐Carbomer; AVC, D‐PA‐AVC, indirect plasma activated AVC (I‐PA‐AVC) were lyophilized to further qualitatively characterize the chemical structure of hydrogels by the FTIR analysis.^[^ ^68^ ^]^ Physicochemical properties of PAH and PAW were determined using a benchtop ORP meter (METTLER‐TOLEDO, S210‐K), a pH tester and a conductivity meter, respectively. To improve the mobility of PAH and the accuracy of measurement results, the method of pH measurement in “GB/T 13531.1‐2008 General Test Method for Cosmetics”is referred. In detail, one part of hydrogel (accurate to 0.1 g) was weighed and added nine parts of laboratory water (DW) for continuous stirring until well mixed; then their pH and ORP values were measured at room temperature.

### Antibacterial Activity Assay

*Staphylococcus aureus* was used as a model microorganism to evaluate the antibacterial performance of PAH and PAW. By using the gradient dilution inversion plate method, the number of surviving colonies could be accurately counted and the sterilization effect could be characterized by inoculating a series of dilution gradients of the mixed bacterial solution: Individual colonies of *S. aureus* were transferred to 4 mL of TSB liquid medium and incubated overnight at 37 °C. The bacteria culture medium was centrifuged at 5000 rpm for 2 min, and the upper layer of the culture medium was poured off with the following saline washing. The last operation was repeated to obtain a suspension of bacterial cells in sterile saline. Freshly prepared PAW or PAH samples (900 µL) were thoroughly mixed with bacterial suspension (100 µL) and incubated at room temperature for 15 min. The samples were serially diluted 10‐fold with phosphate buffer solution, and 10 µL of the diluted sample was spotted onto TSB agar plates and incubated overnight at 37 °C. Finally, the number of viable bacteria on the agar plates was calculated using the colony‐forming unit method (CFU).

### Analysis of the Roles of ROS and RNS

Five chemical scavengers were used to determine the roles of reactive species in the PAH reactivity^[^ ^69^ ^]^, with the mannitol for ∙OH, sodium azide for ^1^O_2_, and tiron for O_2_^−^, ebselen for ONOO^−^, carboxy‐PTIO for NO. These scavengers were added in PAW with the final concentrations of 100 × 10^−3^, 10 × 10^−3^, 10 × 10^−3^, 1 × 10^−3^, and 100 × 10^−6^ m, respectively, and then rapidly mixed with gel powders. After 10 min of mixing, the PAH with scavengers was mixed with the bacterial solution and incubated as described above. Using the ESR spectroscopy and the enzyme marker to measure the reactive species in hydrogels.

### Statistical Analysis

Statistical analysis and graphing were carried out using OriginPro. Data are from several independent experiments and the experimental results were presented as mean ± standard deviation (SD). Statistical analysis was performed using student's *t*‐test. Asterisks indicate the level of significance as follows: ^*,**,***^ *p*‐values <0.05, <0.01, or <0.001, respectively.

## Conflict of Interest

The authors declare no conflict of interest.

## Supporting information

Supporting Information

## Data Availability

The data that support the findings of this study are available from the corresponding author upon reasonable request.
