# Correction to “Plasma‐Activated Hydrogels for Microbial Disinfection”

**DOI:** 10.1002/advs.202513989

**Published:** 2025-08-22

Jinkun Chen, Zifeng Wang, Jiachen Sun, Renwu Zhou^*^, Li Guo, Hao Zhang, Dingxin Liu^*^, Mingzhe Rong, Kostya (Ken) Ostrikov. Plasma‐Activated Hydrogels for Microbial Disinfection. *Adv. Sci*. 2023, 10, 2207407.

https://doi.org/10.1002/advs.202207407

In the originally published article, Figure 3a contained an error inadvertently. The image of the I‐PA‐AVC freeze‐dried sample was mistakenly applied to the D‐PA‐AVC freeze‐dried sample. The corrected image is presented below.


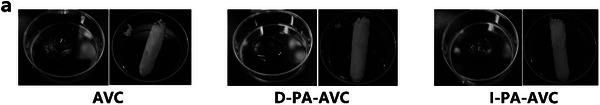


Figure 3. a) Images and freeze‐dried samples of AVC, D‐PA‐AVC, and I‐PA‐AVC.

The authors declare that the correction does not affect the data analysis or conclusions, and they sincerely apologize for the unintentional error.

