# Supplementary material for: Plasma‐Activated Hydrogels for Microbial Disinfection
Source: Adv Sci (Weinh). 2023 Mar 16;10(14):2207407. doi: 10.1002/advs.202207407 (PMC10190649; doi:10.1002/advs.202207407)
Supplement: Supplementary file 1 — Supporting Information [file ADVS-10-2207407-s001.pdf]

Supporting Information

Plasma-activated hydrogels for microbial disinfection

Jinkun Chen[1], Zifeng Wang[1], Jiachen Sun[1], Renwu Zhou[1], Li Guo[1], Hao Zhang[1], Dingxin Liu[1]*, Mingzhe Rong[1], Kostya (Ken) Ostrikov[2]*

Significance, novelty and innovation of this work

We conceptualized and developed a new and very effective way to activate hydrogels using plasmas, turning them into a slow delivery system for short-lived reactive species which are otherwise extremely difficult to control. Only certain types of hydrogels can be activated this way, and this ability is yet largely unknown to both hydrogel and plasma communities.

Conventional hydrogels for antibacterial studies are generally loaded with antibiotics, antimicrobial peptides/polymers or novel metal nanomaterials, but they commonly face several challenges (these challenges are summarized in Table S1). Here we want to emphasize that complementary to the common hydrogel studies, our newly developed plasma-activated hydrogel effectively functions as a carrier and controlled release system of reactive plasma species, with the advantages of no drug resistance, simple preparation method, up to 6 orders of magnitude bactericidal rate, etc. In addition, as a cross-disciplinary study, the novelty of this work lies not only in the development of a novel hydrogel material for the storage and release of otherwise difficult to control short-living radical species, but also in the exploration of the plasma activation method and relevant mechanisms leading to effective microbial disinfection. This knowledge is actively pursued across diverse fields such as soft matter, electrochemistry, plasma physics and chemistry, energy conversion and storage, catalysis, biochemistry, microbiology, and environmental engineering.

- ***The key importance/novelty of this work*** is that we propose a new "plasma-activated hydrogel" (PAH) concept. After the plasma activation, PAH can act as a carrier and delivery system for reactive species that allow effective storage and controlled slow release of RONS to achieve long-term antibacterial effects. Moreover, a detailed comparative analysis of the physico-chemical properties of direct and indirect plasma-activated hydrogels, an investigation of the long-term antimicrobial activity of plasma-activated hydrogel, and the validation of the important effects of short-lived $ONOO^-$,

O_2^-, $^1O_2$, and ·OH species leading to the significant antimicrobial activity of PAH are demonstrated..

Further innovative insights achieved in our work are:

- In this study, for the first time, we discovered that PAH prepared by the plasma activated water (PAW) possessed stronger antimicrobial activity than the commonly used PAW. This is another important supportive evidence that plasma-activated hydrogel indeed act as an effective carrier for reactive plasma species.
- Various short-lived reactive species were quantitatively detected in PAH, and their individual contributions to the antimicrobial activity were identified. These findings can be used by other researchers as a basis for future plasma chemistry modulation and PAH preparation for diverse biomedical and environmental applications.
- In addition, to further optimize the synthesis process of PAH and to increase the real value of PAH for practical applications, we also studied in detail the effects of different gel types and activation methods on the antimicrobial effects of PAH.
- The controlled storage and release of short-lived radicals in PAH can be exploited well beyond microbial inactivation which was used as a representative proof-of-principle application of the new hydrogel-based materials system and mechanisms of radical species confinement and release.

Table S1. Advantages and disadvantages of conventional hydrogel approach and our developed PAH approach for microbial inactivation

Technique	Example	Advantages	Disadvantages	Ref.
Antibiotic-loaded hydrogels	• The self-assembly of a weakly soluble antibiotic (ciprofloxacin) and a hydrophobic tripeptide (DLeu-Phe-Phe) into supramolecular nanostructures that yield a macroscopic hydrogel at physiological pH	Antibiotics are effective against bacterial infections and possess long-lasting antibacterial activity	Leading to the emergence of multi-drug-resistant bacteria	S1-S4
		Capable of slow release at specific application sites	Antibiotics may induce toxic side effects on tissues	
Antimicrobial peptide/polymer - loaded hydrogel	• Used oxidized konjac glucomannan (OKGM), γ-poly(glutamic acid) modified with dopamine and L-cysteine (γ-PGA-DA-Cys) and ε-polylysine (ε-PL) to produce an OKGM/γ-PGA-DA-Cys/ε-PL (OKPP) hydrogel<br>• Mixing polydextran aldehyde and branched polyethylenimine	High biocompatibility and promoting wound healing	Low sterilization efficiency	S4-S6
		Bonding wounds and inhibiting bacterial growth	Part of the material itself or polymerization is not completely toxic	
		No drug resistance	Complex preparation process, immature process	
Metal nanoparticle-loaded hydrogels	Using Mimosa tenuiflora (MtE) extracts as reducing agents to synthesize silver nanoparticles (AgMt NPs). Carbopol hydrogels were made, and the MtE and the synthesized AgMt NPs were dispersed in different gels (MtE-G and AgMt NPs-G, respectively) at 100 μg/g concentration	Broad-spectrum antibacterial properties	Presence of cytotoxicity	S7-S9
		Functions that respond to signals such as light, magnetic flux, and mechanical stimuli	Low biocompatibility	
			Nanoparticles are unstable in terms of physicochemical properties	
Plasma-activated hydrogels	Plasma-activated acryloyldimethylammonium taurate / VP copolymer (AVC) hydrogels	A medium for effective storage and release of short-lived reactive plasma species, insignificant drug resistance	Cross-linking of reactive species and polymers is not fully studied	This work
		Simple preparation process and low cost	Mechanical properties require further improvement for real-world applications	
		High sterilization efficiency		

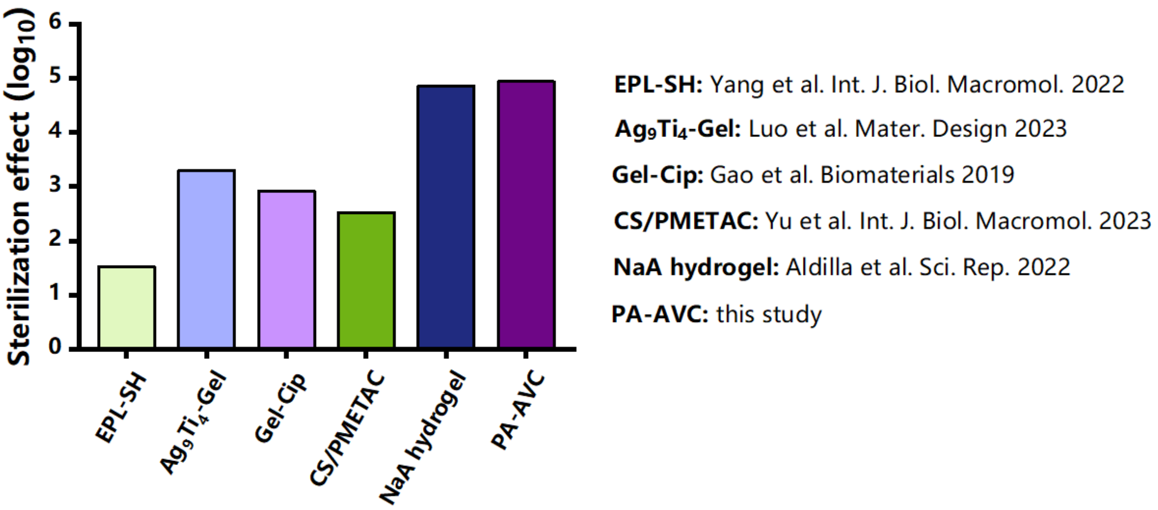

Figure S1. Comparison of the sterilization effect of different types of hydrogels on *S. aureus* obtained from the previous studies and this work.

Table S1 lists the characteristics of different types of antimicrobial hydrogels, however there is a wide range of hydrogels and different methods used to evaluate the antimicrobial effect. In Figure S1, antimicrobial hydrogels (loaded with antibiotics, antimicrobial peptides/polymers or advanced metal-based nanomaterials, etc.) with *S. aureus* as the antimicrobial strain and with quantifiable sterilization effects in relevant studies have been selected for the comparative analysis.

EPL-SH: a multifunctional hydrogel based on polylysine-*graft*-cysteine (EPL-SH) / oxidized dextran (ODex) [S10];

Ag_9Ti_4-Gel: Ag-Titanium-oxo-cluster (Ag-TOC, Ag9Ti4) was developed with active site Ag and salicylic acid, introduced into the dopamine-containing hydrogel system [S11];

Gel-Cip: The drug reservoir was fabricated by mixing ciprofloxacin (Cip, a potent antibiotic) -loaded polydopamine (PDA) nanoparticles (NPs) and glycol chitosan (GC) to form an injectable hydrogel (PDA NP-Cip / GC hydrogel, abbreviated as Gel-Cip) [S12];

CS/PMETAC: a multifunctional CS / Poly [2 - (methacryloyloxy) ethyl] trimethyl ammonium chloride (PMETAC) hydrogel [S13];

NaA hydrogel: the hydrogels prepared through the self-assembly of naphthyl anthranilamide (NaA) capped amino acid based cationic peptide mimics [S6];

PA-AVC: plasma-activated acryloyldimethylammonium taurate / VP copolymer (AVC) hydrogels (this work);

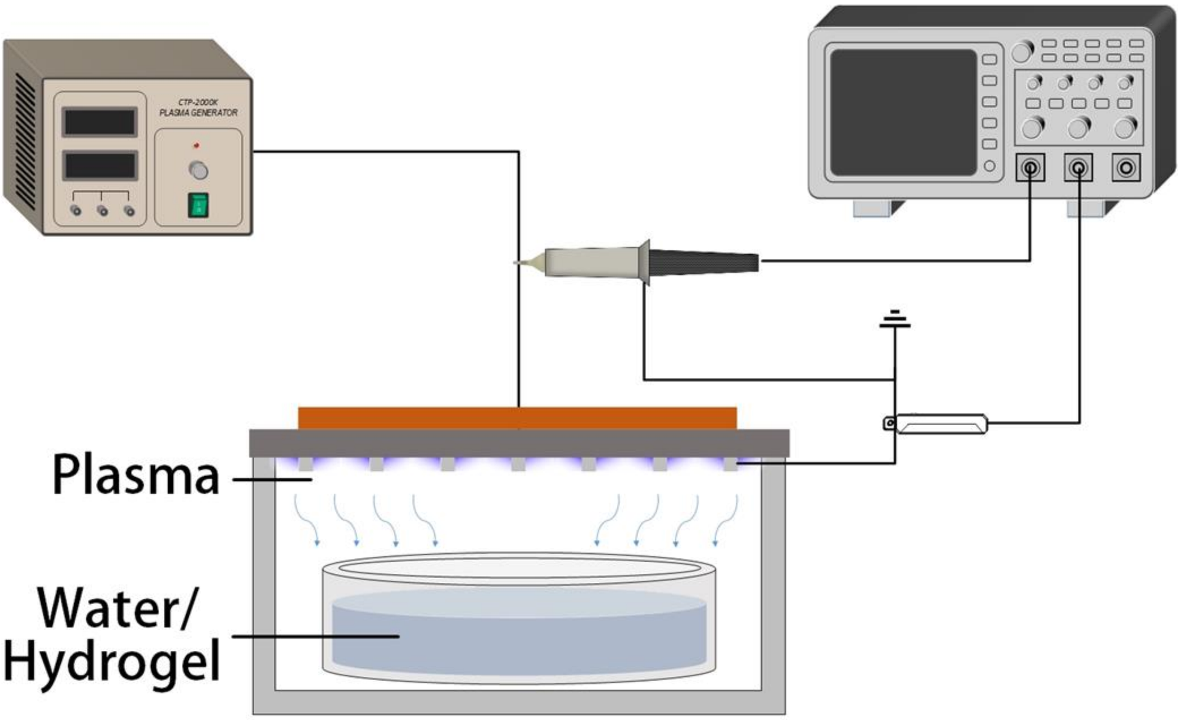

Figure S2. Experimental wiring diagram.

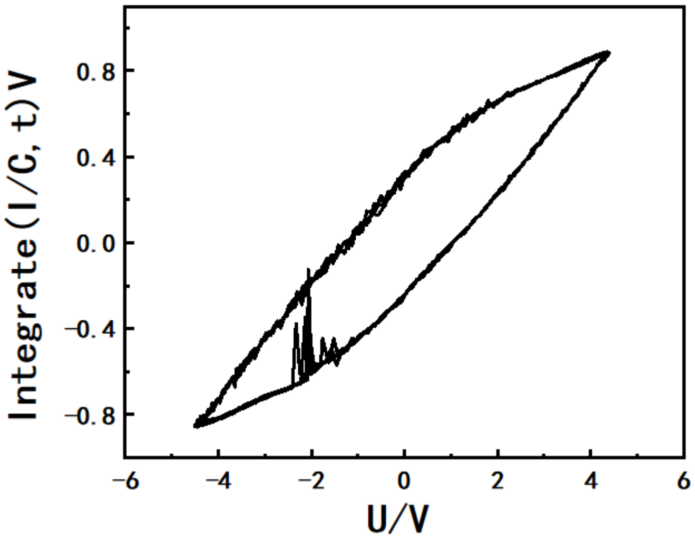

Figure S3. Lissajous figure of surface plasma discharge.

The area of the Lissajous figure is calculated by integration and brought into the formula,

$$Pout=f\times C\times k\times k_x\times k_y\times S$$

the output power (Pout) of the power supply (plasma generator discharge power) can be calculated, where f is the voltage frequency, C is the sampling capacitance, k is the voltage sampling ratio, k_x, k_y are the CH_1,CH_2 signal acquisition attenuation multiplier.

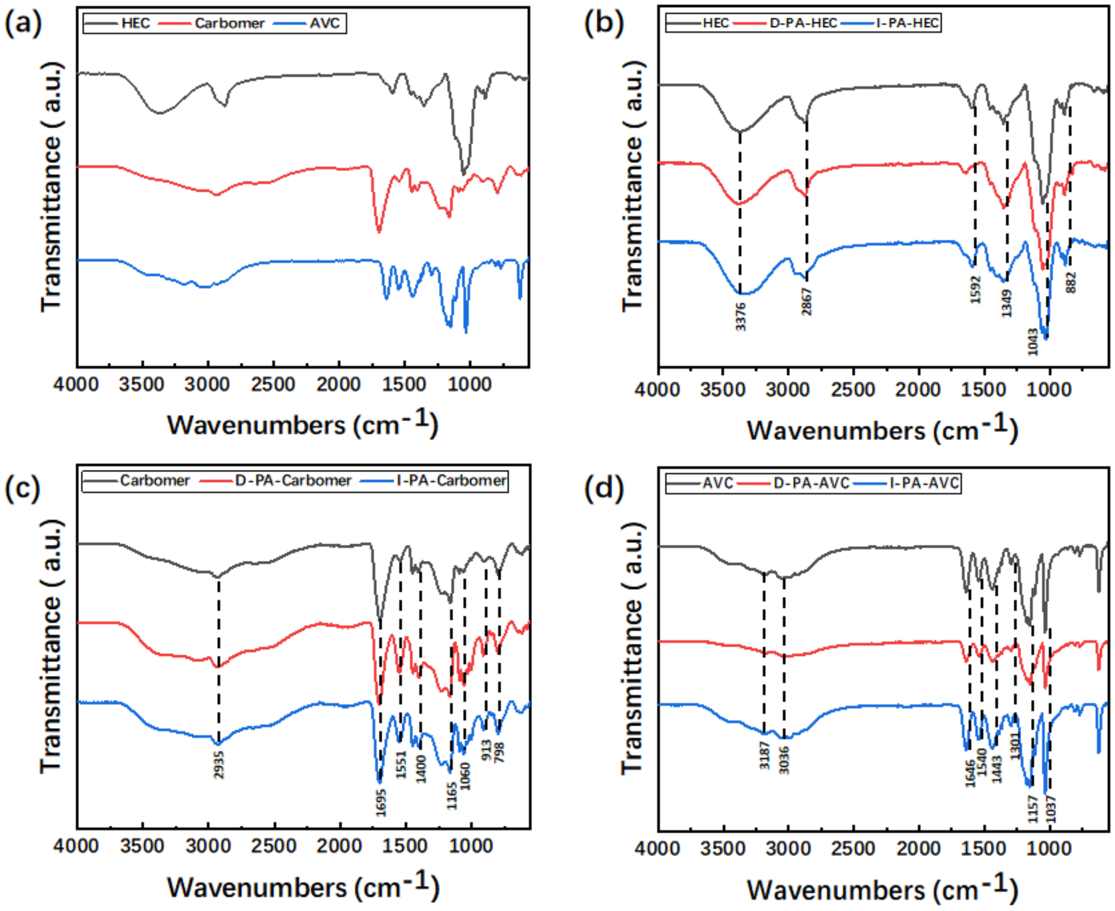

Figure S4. FTIR spectra of three model bio-gels.
(a) FTIR spectra of three hydrogels (HEC, Carbomer, AVC) without the plasma treatment;
(b) FTIR spectra of HEC, D-PA-HEC and I-PA-HEC;
(c) FTIR spectra of Carbomer, D-PA-Carbomer and I-PA-Carbomer;
(d) FTIR spectra of AVC, D-PA-AVC and I-PA-AVC.

The absorption of infrared light at specific wavelengths when the molecules of a compound vibrate produces characteristic absorption peaks, which enable the identification of the surface groups of the hydrogel. For instance, the absorption peaks of HEC were more obvious around 3376 cm^{-1} (O-H stretching vibration) and 2867 cm^{-1} (-CH_2- stretching vibration); the absorption peaks of Carbomer were more obvious around 2935 cm^{-1} (O-H stretching vibration) and 1695 cm^{-1} (C=O stretching vibration); the absorption peaks of AVC were more obvious around 3187 cm^{-1} (N-H stretching vibration), 3036 cm^{-1} (-C=C-H stretching vibration), and 1646 cm^{-1} (-C=C-stretching vibration). However, no significant absorption peak shifts were observed in the FTIR spectra of the three gels after the plasma treatment.

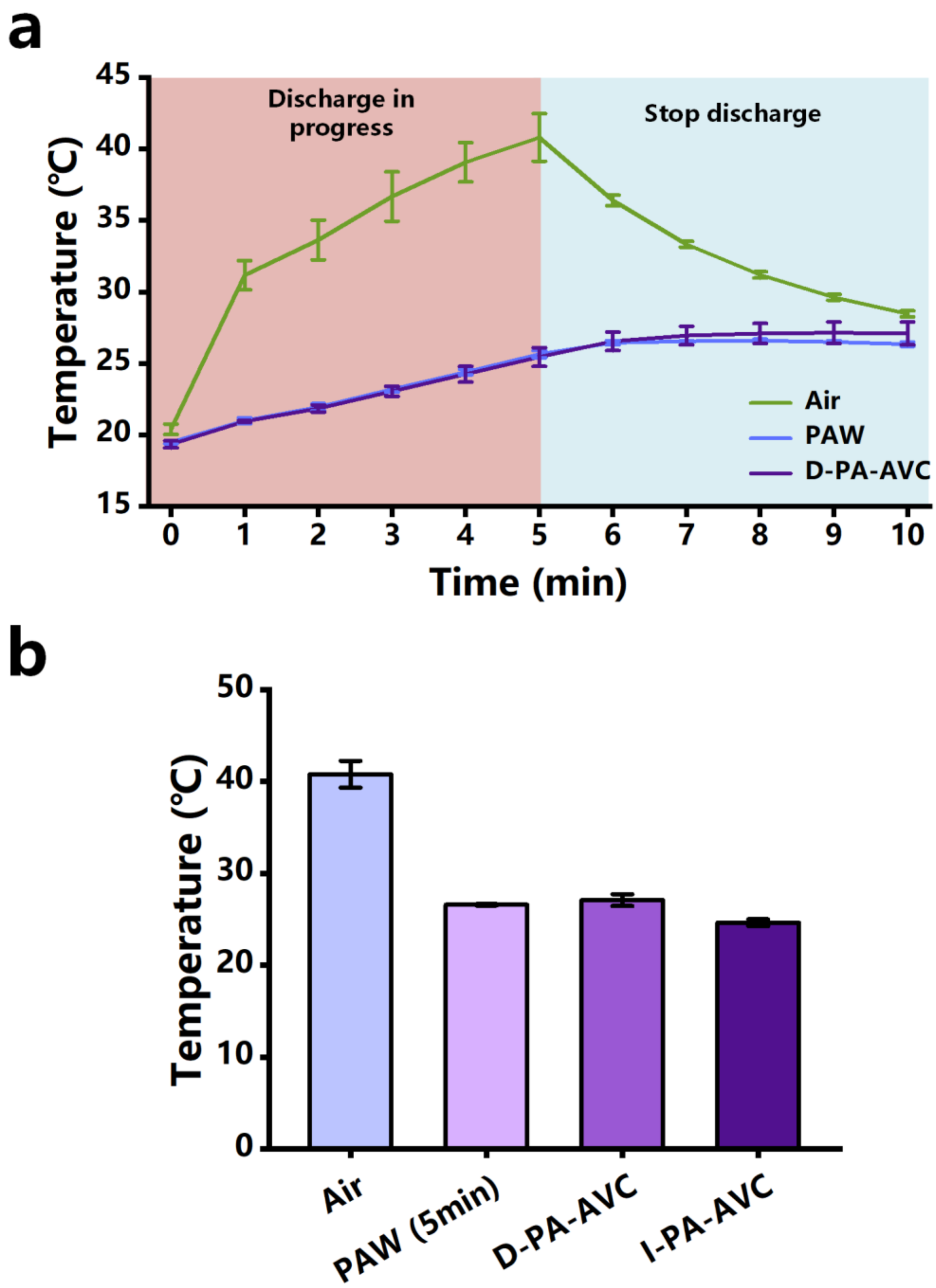

Figure S5. Temperature monitoring of Air, PAW and D-PA-AVC during PAH preparation. (a)Temperature fluctuations of Air, PAW and D-PA-AVC at 5min and 5min after discharge (b)Maximum temperature for Air, PAW, D-PA-AVC and I-PA-AVC

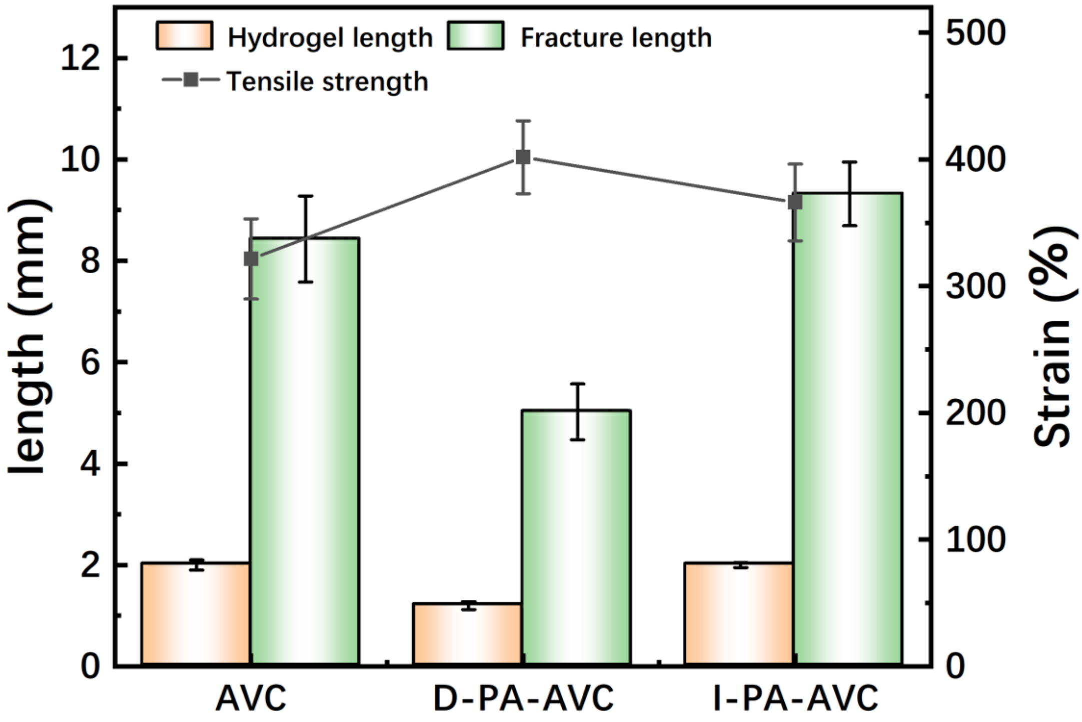

Figure S6. Tensile length and strain of three types of AVC, D-PA-AVC and I-PA-AVC.

Although the break length of D-PA-AVC is lower than that of AVC and I-PA-AVC, the gel length of D-PA-AVC is the smallest, so it has the largest Strain (%).

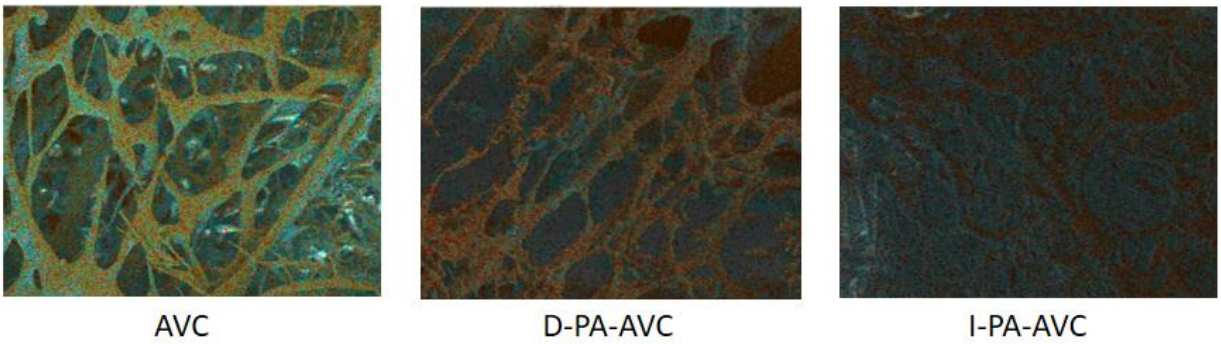

Figure S7. EDS energy spectrum sweep of AVC, D-PA-AVC and I-PA-AVC.

By performing an energy spectrum surface scan of a specified area, it is possible to determine the content of a specific element in that area. A color spot in the graph indicates the distribution of an element within the picture, and the content of an element can be inferred from the density of a color distribution.

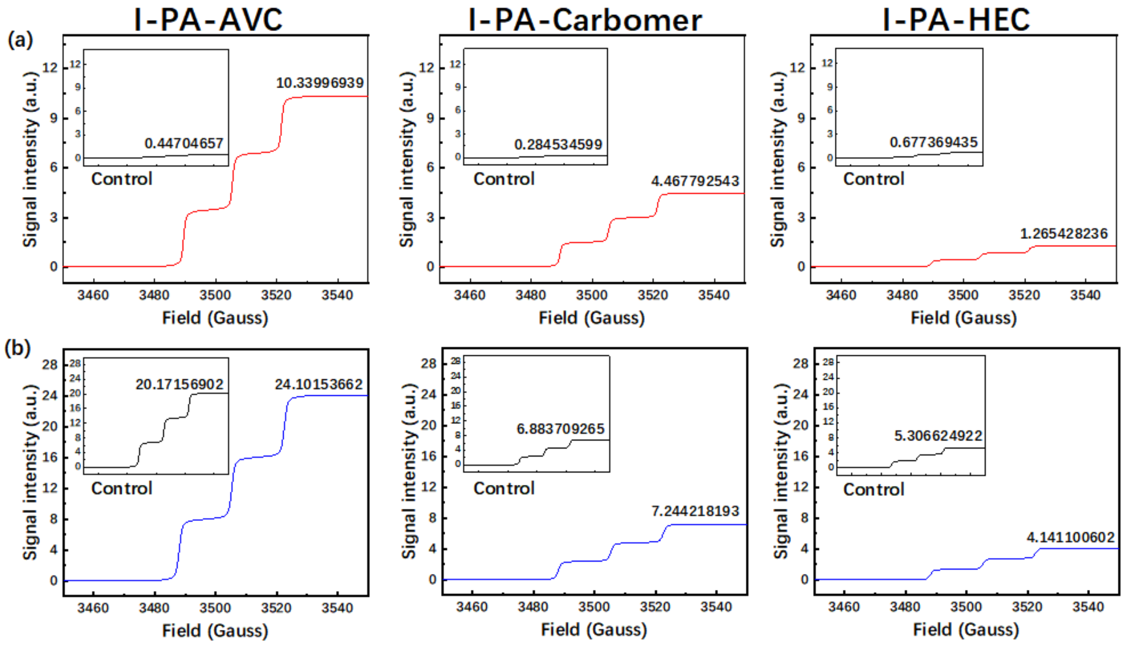

Figure S8. Fitting integral image of ESR spectra about three hydrogels.
(a) Fitting integral image of TEMPONE-H;
(b) Fitting integral image of TEMP.

Figure S7 shows the ESR curve in Figure 5 fitted and integrated, and the integrated value can be used to calculate the concentration of the short-lived reaction species from the nominal curve.

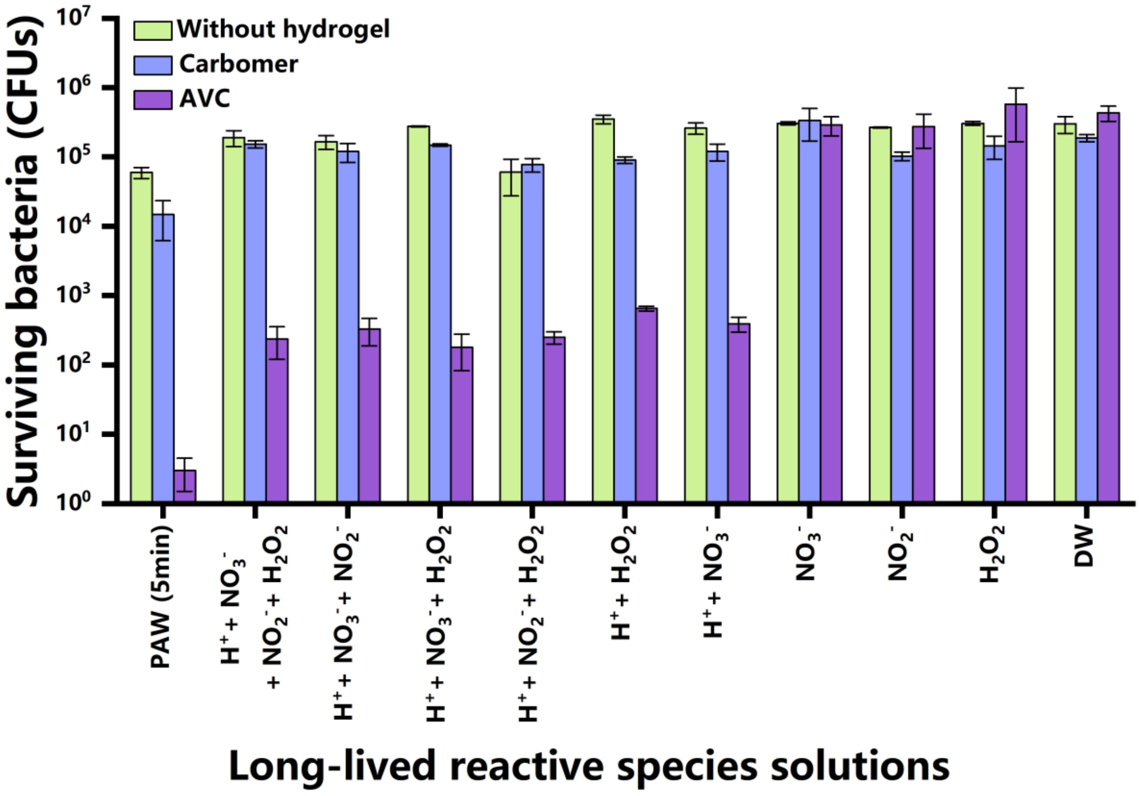

Figure S9. Antimicrobial activity of hydrogels after mixing Carbomer, AVC and long-lived reactive species solutions

The names of the horizontal coordinates in the figure are the long-lived reaction species solutions we configured, and the concentrations of the species remain approximately the same as those measured in the PAW in Figure 1h and 1i. This method can be used to explore the antimicrobial activity of PAH when only long-lived reactive species are available.

References:

[S1] M. Fu, Y. Gan, F. Jiang, X. Lv, N. Tan, X. Zhao, Y. Yang, P. Yuan, X. Ding, Adv. Healthc. Mater., 2022, 11(15), 2200902.

[S2] O. C. J. Andrén, T. Ingverud, D. Hult, J. Håkansson, Y. Bogestål, J. S. Caous, K. Blom, Y. Zhang, T. Andersson, E. Pedersen, C. Björn, P. Löwenhielm, M. Malkoch, Adv. Healthc. Mater., 2019, 8, 1801619.

[S3] S. Marchesa., Y. Qu, L. J. Waddington, C. D. Easton, V. Glattauer, T. J. Lithgow, K. M. McLean, J. S. Forsythe, P. G. Hartley, Biomaterials, 2013, 34(14), 3678-3687.

[S4] A. Sun, D. Hu, X. H e, X. Ji, T. Li, X. Wei, Z. Qian, NPG Asia Mate., 2022, 14:86.

[S5] M. C. Giano, Z. Ibrahim, S. H. Medina, K. A. Sarhane, J. M. Christensen, Y. Yamada, G. Brandacher, J. P. Schneider, Nat. Commun., 2014, 5:4095.

[S6] V. R. Aldilla, R. Chen, R. Kuppusamy, S. Chakraborty, M. D. P. Willcox, D. StC. Black, P. Thordarson, A. D. Martin, N. Kumar, Sci. Rep., 2022, 12:22259

[S7] A. Martínez-Higuera, C. Rodríguez-Beas, J. M. A. Villalobos-Noriega, A. Arizmendi-Grijalva, C. Ochoa-Sánchez, E. Larios-Rodríguez, J. M. Martínez-Soto, E. Rodríguez-León, C. Ibarra-Zazueta, R. Mora-Monroy, H. A. Borbón-Nuñez, A. García-Galaz, M. del, C. Candia-Plata, L. F. López-Soto, R. Iñiguez-Palomares, Sci. Rep., 2021, 11:11312.

[S8] A. J.Clasky, J. D.Watchorn, P. Z.Chen, F. X. Gu, Acta Biomater., 2021, 122, 1-25.

[S9] S. Li, S. Dong, W. Xu, S. Tu, L. Yan, C. Zhao, J. Ding, X. Chen, Adv. Sci., 2018, 5(5), 1700527.

[S10] R. Yang, W. Xue, H. Liao, F. Wu, H. Guo, W. Zhang, P. Wang, X. Tan, H. Xu, B. Chi, Int. J. Biol. Macromol., 2022, 223, 950-960.

[S11] W. Luo, B. Hu, H. Zhang , C. Li, Y. Shi, X. Li, L. Jin, Mater. Design, 2023, 226, 111674.

[S12] G. Gao, Y. Jiang, H. Jia, F. Wu, Biomaterials, 2019, 188, 83-95.

[S13] Q. Yu, Y. Yan, J. Huang, Q. Liang, J. Li, B. Wang, B. Ma ,A. Bianco, S. Ge, J. Shao, Int. J. Biol. Macromol., 2023, 231, 123149.
